# Testing the performance of polygenic scores for multiple traits to explain cerebral palsy in two independent cohorts

**DOI:** 10.1016/j.ebiom.2026.106208

**Published:** 2026-03-14

**Authors:** Jodi T. Thomas, Alexander S.F. Berry, Matthew T. Oetjens, Jesia G. Berry, Alastair H. MacLennan, Scott D. Gordon, Andrew T. Hale, Catherine M. Olsen, David C. Whiteman, Rebecca I. Torene, David H. Ledbetter, Nicholas G. Martin, Clare L. van Eyk, Jozef Gecz, Scott M. Myers, Brittany L. Mitchell, Mark A. Corbett

**Affiliations:** a Brain and Mental Health Program, QIMR Berghofer, Brisbane, QLD, Australia; b School of Biomedical Sciences, The University of Queensland, Brisbane, QLD, Australia; c Department of Developmental Medicine, Geisinger College of Health Sciences, Lewisburg, PA, USA; d School of Medicine and Robinson Research Institute, College of Health, Adelaide University, Adelaide, SA, Australia; e Department of Neurosurgery, The University of Alabama at Birmingham, Birmingham, AL, USA; f Neuroscience Institute, University of Cape Town, Cape Town, South Africa; g Population Health Program, QIMR Berghofer, Brisbane, QLD, Australia; h Institute for Pediatric Rare Diseases, College of Medicine, Florida State University, Tallahassee, FL, USA; i South Australian Health and Medical Research Institute, Adelaide, SA, Australia; j School of Biomedical Sciences, Faculty of Health, Queensland University of Technology, Brisbane, QLD, Australia

**Keywords:** Cerebral palsy, Hypoxic ischaemic encephalopathy, Non-progressive movement disorder, Intellectual disability, Stroke, Genome wide association study, Polygenic

## Abstract

**Background:**

Cerebral palsy (CP) is a complex neurodevelopmental disorder with both environmental and genetic contributors. Rare genetic variants explain a substantial proportion of CP, but the contribution of common variants remains unclear. We evaluated whether polygenic scores for CP and related traits explain the aetiology of CP.

**Methods:**

We analysed two independent target cohorts: a case–control cohort including people with a confirmed clinical diagnosis of CP from the Australian CP Biobank and population-based controls; and MyCode, a United States healthcare cohort with CP status identified by electronic health records. Only participants of European genetic ancestry were included. CP polygenic scores were constructed using a publicly available discovery genome-wide association meta-analysis of Finnish and UK cohorts (n_cases_ = 624, n_controls_ = 495,687) and applied to the target cohorts for out-of-sample prediction. Additional polygenic scores were generated from publicly available genome-wide association studies for seven CP-related traits. Predictive performance was assessed using logistic regression, area under the receiver operating characteristic curve, and variance in CP liability explained.

**Findings:**

The Australian cohort included 525 cases and 20,410 controls, and MyCode 322 cases and 1610 age-matched controls. The combined model of all eight polygenic scores significantly discriminated CP status, explaining 1·3% of CP liability in the Australian cohort (90% CI lower bound 0·82%, padj<0·0001), and 0·78% in MyCode (90% CI lower bound 0·35%, padj<0·0001). CP-specific polygenic scores demonstrated minimal predictive signal, likely reflecting limited GWAS power. Polygenic scores for known CP predisposing factors (birth weight, gestational duration, stroke) showed modest predictive performance, with some cohort differences. Results were similar when the Australian cohort was stratified by monogenic CP diagnosis.

**Interpretation:**

Our findings demonstrate a measurable polygenic contribution to CP and shared genetic influences with predisposing factors, including those traditionally considered environmental, and comorbidities. Common variants appear to contribute broadly to CP susceptibility, highlighting a multifactorial landscape relevant for earlier diagnosis and intervention.

**Funding:**

Cerebral Palsy Alliance Research Foundation, NICHD, NHMRC, MRFF, QIMR Berghofer.

**Research in context**

**Evidence before this study**

We searched PubMed from database inception to September 2, 2025, with no language restrictions, for peer-reviewed articles on cerebral palsy (CP) that assessed the contribution of common variants to CP aetiology, either individually or through a polygenic basis. The search terms used were (“cerebral palsy”) AND (“genome-wide association study” OR “genome-wide association” OR “GWAS” OR “genetic association” OR “polymorphism” OR “polygenic” OR “polygenic risk” OR “polygenic score” OR “common varia∗”). The search identified 123 articles, of which 49 were excluded as not relevant following manual review of the abstracts because they did not evaluate CP in the context of genetic association. Most studies assessed the association of individual single nucleotide polymorphisms (SNPs) with CP outcomes even when multiple SNPs were examined within the same study, rather than considering their combined effects. Systematic reviews and meta-analyses conducted to date have found potential genetic associations with CP, CP severity, or gene environment interactions between candidate SNPs and known CP predisposing factors, such as susceptibility to viral infection. Candidate SNPs were selected based on known CP predisposing factors or biological pathways, such as genes implicated in thrombophilia (e.g., *F5, F2, MTHFR*), infection and inflammation (e.g., *TNF, IL6, LTA*), and ischaemic stroke (e.g., *APOE, COL4A1, COL4A2*). A single genome-wide association study (GWAS) of CP has been published, which identified a single genome-wide significant variant. Additionally, GWAS summary statistics from a meta-analysis combining FinnGen and UK Biobank data are publicly available through the FinnGen consortium. Although no peer-reviewed manuscript has been published for this meta-analysis, it represents, to our knowledge, the largest GWAS of CP conducted to date, and has not identified any genome-wide significant variants. Polygenic aetiology has been evaluated on a limited basis in CP using selected variants in genes implicated in dopamine transmission, rather than genome-wide. These studies found a potential association with training performance in children with CP, and a relationship to poorer psychomotor development that was not correlated with a CP diagnosis. Most recently, Mendelian Randomisation studies using CP GWAS data as the outcome suggest that polygenic liability to gestational diabetes or to complement-pathway protein levels may have causal links to CP. However, no study has yet used CP GWAS data to quantify the genome-wide polygenic contribution to CP, or to perform independent genome-wide polygenic analyses.

**Added value of this study**

Using publicly available GWAS data, we constructed polygenic scores for CP and seven related traits (age at walking onset, birthweight, gestational duration, stroke, autism spectrum disorder, epilepsy, and educational attainment) and tested their predictive potential in two independent cohorts. We demonstrate that CP has a measurable polygenic basis that is shared with common neurodevelopmental comorbidities and prenatal predisposing factors, including those traditionally considered environmental. These effects were consistent regardless of the presence of a monogenic diagnosis, indicating that common variants may contribute to CP across the spectrum of aetiologies.

**Implications of all the available evidence**

Our findings provide strong support for a polygenic contribution to CP aetiology. Such knowledge may help explain phenomena such as pleiotropy in families where multiple neurodevelopmental traits, such as CP, epilepsy, autism, and intellectual disability co-occur. Polygenic liability could also influence recovery from environmental causes of CP and shape responses to treatment and prevention strategies. These results highlight the need for large international collaborations to increase CP GWAS sample sizes and improve the precision of polygenic scores. In the longer term, such scores could be integrated with rare variant and clinical data to improve early detection, diagnosis, and timely intervention, ultimately supporting better health outcomes for people living with CP.

## Introduction

Cerebral palsy (CP) is an early-onset, lifelong condition caused by a nondegenerative maldevelopment of the fetal or infant brain that leads to limitations in movement and posture.^1^^,^^2^ CP is an umbrella term that captures a heterogeneous spectrum of clinical conditions. Approximately half of individuals living with CP may also have intellectual impairments, two thirds have speech impairments, a third have epilepsy, a third have vision impairments, and up to a third live with autism spectrum disorders (ASD).^3^^,^^4^ The prevalence of CP varies throughout the world, but is approximately double in low-middle income countries compared to high income countries.^5^ The birth prevalence of CP is approximately 1·3–1·6 and 2–4 per 1000 live births in Australia and the United States, respectively.^5^^,^^6^

The aetiology of CP was traditionally regarded as environmental, however incidence of CP is higher in individuals with consanguineous parents, those with a family history of CP, and has greater concordance in monozygotic compared to dizygotic twins which suggests a significant genetic contribution.^7^, ^8^, ^9^, ^10^ Multiple clinical exome and genome sequencing studies have shown that rare variants underlying a monogenic cause account for 25–31% of CP (or 17·6% when excluding individuals with intellectual impairment or developmental delay).^11^^,^^12^ Furthermore, the monogenic causes of neurodevelopmental comorbidities such as intellectual disability, epilepsy and ASD show significant overlap with genes implicated in CP.^13^

Combinations of common genetic variants, each with a small effect, may also contribute to CP aetiology (i.e., polygenic aetiology), however this has not been comprehensively tested. Early evidence for common variant involvement in CP has largely come from candidate single nucleotide polymorphism (SNP) association studies. A systematic review of these studies indicated CP was likely to be associated with SNPs in the genes Factor V Leiden (*F5*), methylenetetrahydrofolate reductase (*MTHFR*), Tumour necrosis factor alpha (*TNF*), Lymphotoxin alpha (*LTA*), Nitric oxide synthase 3 (*NOS3*, a.k.a. eNOS) and Mannose binding lectin (*MBL2*).^14^ However, associations reported in candidate gene studies should be interpreted with caution as most studies were small, hypothesis-driven and shown to be at risk of type I and II errors.^14^ A subsequent meta-analysis of 17 candidate SNPs from 11 association studies that encompassed 2533 cases and 4432 controls found a robust association between the SNP rs1800795 in the *IL6* gene and CP; however, a polygenic score was not evaluated.^15^ Of the two genome-wide association studies (GWAS) conducted to date, one identified one significant locus,^16^ while a larger meta-analysis combining FinnGen and the UK Biobank, did not identify any significant loci (https://public-metaresults-fg-ukbb.finngen.fi/pheno/G6_CP).^17^ No previous studies have quantified the genome-wide polygenic contribution to CP or performed polygenic analyses.

Polygenic scores (PGS), which use GWAS results and sum the effects of common variants weighted by their contribution to CP aetiology, could be a powerful complement to magnetic resonance imaging (MRI), clinical signs, and monogenic testing to improve early prediction of CP outcomes.^11^^,^^18^ This is crucial as early detection provides the best opportunity for therapeutic and physical interventions for long-term improvement in outcomes.^19^^,^^20^ Assessment of polygenic scores using existing CP GWAS is likely to be underpowered, however many neurodevelopmental comorbidities of CP (e.g., epilepsy, ASD, intellectual disability) and predisposing factors for CP traditionally considered environmental (e.g., prematurity, birth complications, foetal growth restriction) also have a polygenic basis that could be partially shared with CP.^21^, ^22^, ^23^, ^24^, ^25^, ^26^ Leveraging polygenic scores from both CP and these related traits could therefore enhance predictive power and provide insight into shared genetic architecture.

In this study, we aimed to evaluate the predictive ability of polygenic scores for CP to gain insight into the potential polygenic architecture of CP. Given the limited power of CP GWAS to date, we incorporated polygenic scores constructed from large GWAS of related traits to increase predictive power. We also sought to determine whether polygenic score performance differs between individuals with and without a monogenic diagnosis. This work provides the foundation for future assessment of the utility of polygenic scores for early CP detection and diagnosis.

## Methods

### Study design

The Australian case–control cohort included individuals with cerebral palsy (CP) from the Australian CP Biobank (2010–2024), and population-based controls from the QSkin Sun and Health Study (QSkin) (2010–2011 and 2019–2020).^27^ Ethics approval was obtained from the Women's and Children's Health Network ethics committee for the Australian CP Biobank (HREC/12/WCHN/61 and HREC/15/WCHN/148), and from the QIMR Berghofer Medical Research Institute Human Research Ethics Committee for QSkin (P1309, P2034, and P3434) and the combined analysis (P3964).

The United States (U.S.) healthcare cohort used data from the MyCode Community Health Initiative (January 1 2005–December 31 2023).^28^ The study was approved by the Geisinger Institutional Review Board (IRB).

The study adheres to the ethical principles of the World Medical Association 2024 Declaration of Helsinki.

### Participants

#### Australian cohort

Individuals were recruited into the Australian CP Biobank through hospital-based CP services (paediatric rehabilitation and/or orthopaedics), supplemented by specialist referrals and community outreach. Clinical data were sourced from questionnaires and medical records. CP diagnoses were confirmed at or after four years of age by a neurologist or paediatric rehabilitation specialist, consistent with international standards.^2^ Individuals with postnatally acquired CP were excluded. Controls were defined as all participants from QSkin, a population-based cohort from Queensland, Australia, recruited via a random electoral-roll sample.^27^ The overall study size was determined by the number of eligible participants recruited within the study timeframe in each cohort. Although QSkin participants were not individually screened for CP, given the low prevalence of CP, any inadvertent inclusion of individuals with CP is expected to have a negligible impact. Informed consent was obtained from all participants or their legal guardians.

#### MyCode

MyCode participants are primarily adults, with informed consent obtained from adults and from parents or guardians of paediatric participants.^28^ CP diagnosis were assigned by treating clinicians and extracted from electronic health records (EHR) using either ICD-9 codes (333·71, 343·0, 343·1, 343·2, 343·3, 343·8, 343·9) or ICD-10 codes (G80·0, G80·1, G80·2, G80·3, G80·4, G80·8, G80·9). Participants were identified through a rule-based approach requiring at least two relevant codes, either repeated or distinct, from inpatient or outpatient encounters. To reduce misclassification from provisional or rule-out diagnoses, codes appearing only in billing records were excluded. Individuals with only one CP code underwent manual chart review for confirmation. Controls had no CP codes and were excluded if they had ever been seen by the Geisinger Department of Developmental Medicine to minimise the risk of including people with undiagnosed CP. The sample size reflected all eligible participants recruited during the study timeframe.

### Procedures

#### Polygenic scores

**Fig. 1:**
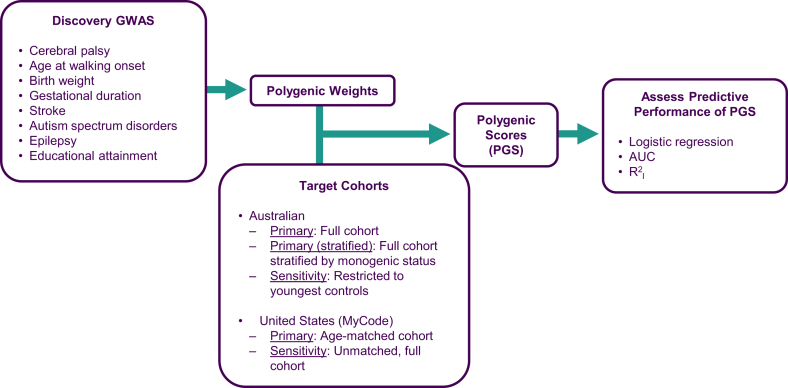
Workflow for polygenic score analysis. Discovery GWAS summary statistics for CP and seven related traits were used to generate polygenic weights. These traits included a trait related to motor development, and known CP predisposing factors and comorbidities with sufficiently powered GWAS (≥5 independent genome-wide significant variants). Polygenic weights were applied to two independent target cohorts to calculate individual polygenic scores for each trait. Polygenic scores predictive performance was evaluated using logistic regression, area under the receiver operating characteristic curve (AUC), and variance explained in CP on the liability scale (R^2^_l_). The primary analysis used the full Australian cohort and the age-matched MyCode cohort. Another primary analysis was only conducted in the Australian cohort and stratified the cohort by the presence or absence of a monogenic diagnosis for CP. A sensitivity analysis included the youngest 525 Australian controls (1:1 with cases) to assess age bias, and the full, unmatched MyCode cohort.

Polygenic scores (PGS) were constructed from publicly available genome-wide association study (GWAS) summary statistics for CP and seven related traits; age at walking onset (motor development),^29^ predisposing factors traditionally considered environmental (birth weight,^26^ gestational duration,^24^ any type of stroke^22^^,^^30^), and comorbidities (autism spectrum disorder (ASD),^21^ epilepsy,^25^ and educational attainment (EA) as a proxy for intellectual disability^23^) (Fig. 1, Table 1). GWAS summary statistics were used in SBayesRC in GCTB (v2·5·2)^31^ to create polygenic weights, which were then applied to individual genotype data from each target cohort to construct PGS using PLINK (v1·9).^32^ Only individuals of European genetic ancestry were included. See Supplementary Text A for more details on genotyping, quality control, and polygenic scores.

**Table 1:** Overview of traits for which polygenic scores were constructed.

Trait	N	Overlap/exclusions	Rationale	GWAS Reference
Cerebral Palsy (FinnGen phenotype G6_CP: ICD-10 code G80 and ICD-8 or 9 code 343)	3465		Directly tests polygenic contribution to CP. CP inclusion criteria is similar to MyCode.	Kurki et al.^17^^,^^a^
Age at walking onset	70,560		Captures motor development, a core domain affected in CP	Gui et al.^29^
Birth Weight	298,142		A person with CP is seven times more likely to be born small for their gestational age than the population average	Warrington et al.^26^
Gestational Duration	151,987	Excluding 23andMe, Inc., data	A person with CP is four times more likely to have had a preterm birth than the population average	Solé-Navais et al.^24^
Stroke	278,028	For Australian polygenic scores	At least 30% of neonates affected by perinatal stroke will develop CP	Mishra et al.^22^
	147,590	For MyCode polygenic scores (used because MyCode data are included in Mishra et al.^22^)		Malik et al.^30^
Autism spectrum disorders	44,367		Comorbidity with 8%–30% of people living with CP	Grove et al.^21^
Epilepsy	65,124		Comorbidity with approximately 30% of people living with CP	Stevelink et al.^25^
Educational Attainment	757,277	Excluded QIMR data (Australian polygenic scores)	Proxy for cognitive function/intellectual disability, which affects around 50% of people with CP	Okbay et al.^23^
	750,721	Excluded MyCode data (MyCode polygenic scores)		
		All excluded 23andMe, Inc., data		

For each trait, details of the genome-wide association study (GWAS) used are displayed, including sample size (N), reported as effective sample size for binary traits and as a total sample size for quantitative traits. Effective sample size was calculated using 4/(1/[number of cases] + 1/[number of controls]). The overlap/exclusions column describes steps taken to avoid sample overlap between the GWAS discovery data and the Australian and MyCode cohorts, thereby preventing bias and ensuring independent predictions. Data from 23andMe, Inc. were excluded due to lack of approval for use and this is also indicated where applicable in the Overlap/exclusions column. The Rationale column provides information for why each trait was included, and the GWAS Reference column cites the corresponding publication for the GWAS used. For all traits, the GWAS summary statistics from analysis including Europeans only were used.

a CP GWAS summary statistics provided by FinnGen; FinnGen release 12 meta-analysed with Europeans from the UK Biobank (GWAS meta-analysis results: https://public-metaresults-fg-ukbb.finngen.fi/pheno/G6_CP and methods: https://finngen.gitbook.io/documentation/methods/meta-analysis).

### Statistical analysis

PGS for CP and seven related traits were evaluated for their ability to distinguish CP status in the Australian and MyCode cohorts, as well as in the Australian cohort stratified by monogenic diagnosis (Fig. 1). A monogenic diagnosis (presence of a known rare variant for CP) was inferred from exome, genome or CP gene panel sequencing as previously described.^33^, ^34^, ^35^ Relatedness was assessed using PLINK (v1·9),^32^ and one individual per related pair (kinship coefficient >0·09) was removed, preferentially from the controls, while retaining the maximally sized cohort.

Predictive performance was assessed using 1) logistic regression, 2) area under the receiver operating characteristic curve (AUC), and 3) variance in CP liability explained by the PGS (R^2^_l_). Analyses were conducted in R (Australia: v4·3·1, MyCode: v4·5·1)^36^ using RStudio (Australia: v2024·04·2, MyCode: v2025·05·0).^37^ Significance was defined as an adjusted p-value (Benjamini-Hochberg) < 0·05. PGS were standardised within each cohort so effect sizes reflect a change in CP polygenic score per standard deviation.

For logistic regression, CP status was the outcome. In each cohort, eight single-PGS models were run, and one multiple-PGS model included all eight PGS simultaneously. PGS p-values were adjusted for eight comparisons in both the single-PGS models and multiple-PGS model. Adjusted models (main text) included sex (female/male) and the first 10 ancestry principal components (PCs) as continuous variables; unadjusted models are reported in Supplementary. Sex was inferred from genotype data in the Australian cohort and EHR data in MyCode.

Receiver operating characteristic analyses were conducted with CP status as the outcome using each PGS individually and all PGS combined as the predictors. To test whether the AUC >0·5, Z-scores were calculated with Equation (1), where *x* = AUC, and *H*_*0*_ = 0·5. One-tailed p-values were obtained from the standard normal distribution and adjusted for nine comparisons.

(1) $$Z=\frac{x\;-\;H_{0}}{SE}$$

Variance in CP (R^2^) was estimated using linear regressions with CP status as the outcome. For each PGS, R^2^ was the difference between the full model (PGS + sex +10 PCs) and reduced model (sex +10 PCs). Confidence intervals were derived from 1000 bootstrap samples. Observed R^2^ were converted to the liability scale (R^2^_l_) using the method from Lee et al.,^38^ assuming CP prevalence of 0·0014 (Australia), and 0·0032 (MyCode).^5^^,^^6^ This procedure was applied to single-PGS models and a multiple-PGS model including all eight PGS. To test whether R^2^_l_ > 0, Z-scores were calculated with Equation (1), where *x* = R^2^_l_, *H*_*0*_ = 0. One-tailed p-values were obtained from the standard normal distribution and adjusted for nine comparisons.

#### Sensitivity analyses

We performed sensitivity analyses to assess potential age-related bias. In the Australian cohort, where age-matching was not possible, controls were restricted to the youngest 525 (1:1 with cases). As results were similar, the full cohort is reported as primary and the restricted set as sensitivity. In MyCode, age-matched analyses are reported as the primary analysis, with the unmatched cohort as a sensitivity analysis. Age-matching was performed by matching each person with CP to five controls using the ‘MatchIt’ package v4·7·2 in R.^39^

### Role of the funders

The funders of the study had no role in study design, data collection, data analysis, data interpretation, or writing of the report.

## Results

The Australian cohort recruited 594 individuals with CP (Australian CP Biobank) and 26,465 controls (QSkin). After quality control and excluding related individuals (11 CP and 3492 controls), the final sample comprised 525 people with CP and 20,410 controls. Participants with CP were, on average, 52·33 years younger than controls (95% CI 51·85–52·81, t = 213·01, df = 537·73, p < 0·0001 [two-tailed T-test]), and had fewer females (χ^2^ = 63·80, df = 1, p < 0·0001 [Chi-squared test]). Among those with CP, birthweight was 2491 ± 1095 g, gestational age 35 ± 5 weeks, Gross Motor Function Classification System (GMFCS) scores were skewed toward lower severity, and 23·2% (n = 122) had a monogenic CP diagnosis due to a rare pathogenic or likely pathogenic single nucleotide variant or copy number variant (Table 2). In total, monogenic diagnoses encompassed variants in 103 different genes or genomic loci.

**Table 2:** Sample characteristics for both cohorts included in the analyses.

	Australian Cohort		MyCode	
	Control	CP	Control	CP
Sex (N (%))				
Female	11,750 (57·6%)	210 (40·0%)	1004 (62·4%)	167 (51·9%)
Male	8660 (42·4%)	315 (60·0%)	606 (37·6%)	155 (48·1%)
Unknown	0	0	0	0
Age (years)				
Mean ± SD (range)	61·2 ± 9·6 (18–93)	8·9 ± 5·1 (1–43)	40·6 ± 20·7 (2–89)	40·6 ± 20·8 (2–89)
Unknown	72 (0·4%)	64 (12·2%)	0	0
Birth weight (grams)				
Mean ± SD (range)	N/A	2491·5 ± 1095·0 (218–5550)	N/A	N/A
Unknown	N/A	98 (18·7%)	N/A	N/A
Gestational duration (weeks)				
Mean ± SD (range)	N/A	35·2 ± 5·3 (22·6–42·1)	N/A	N/A
Unknown	N/A	76 (14·5%)	N/A	N/A
GMFCS score (N (%))				
1	N/A	148 (28·2%)	N/A	N/A
2	N/A	127 (24·2%)	N/A	N/A
3	N/A	82 (15·6%)	N/A	N/A
4	N/A	64 (12·2%)	N/A	N/A
5	N/A	39 (7·4%)	N/A	N/A
Unknown	N/A	65 (12·4%)	N/A	N/A
Monogenic diagnosis (N (%))				
Yes	N/A	122 (23·2%)	N/A	N/A
No	N/A	395 (75·2%)	N/A	N/A
Unknown	N/A	8 (1·5%)	N/A	N/A

The Australian cohort used the full set of controls and in MyCode controls were age-matched. Age = age at time of survey (Australia) or age at last electronic health record retrieval (MyCode), CP = cerebral palsy. GMFCS = gross motor function classification, N = sample size, N/A = Data not available. SD = standard deviation. Note that sex is the only variable from this table included in the analyses, all other variables are provided for descriptive purposes.

The U.S. MyCode cohort recruited >350,000 participants, with 172,366 genotyped. After quality control, and excluding 10 participants with unknown sex, 887 missing EHR data, and 26,487 related individuals, the final sample included 129,950 participants. From these, 322 individuals with CP were identified and matched to 1610 controls on age at last EHR retrieval. Participants with CP had fewer females compared to controls (χ^2^ = 11·95, df = 1, p < 0·0001 [Chi-squared test]) (Table 2).

To assess age-related bias, analyses were repeated in the Australian cohort using only the 525 youngest controls, reducing but not eliminating age differences. In MyCode, the unmatched cohort, in which participants with CP were significantly younger than controls, was included as a sensitivity analysis (Supplementary Text B, Supplementary Table S1).

**Fig. 2:**
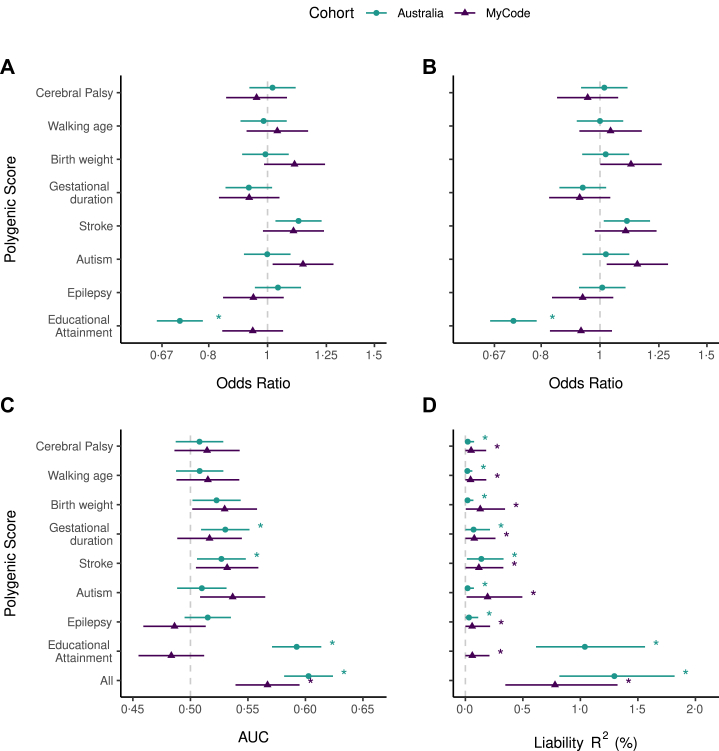
**Ability of polygenic scores for cerebral palsy (CP) and seven related traits to distinguish between individuals with and without CP in two independent cohorts. A)** Single-PGS logistic regressions, adjusted for sex and 10 ancestry PCs. Star = significant association of polygenic score with CP status after p-value adjustment for eight comparisons. **B)** Multiple-PGS logistic regression (all eight polygenic scores in the same model), adjusted for sex and 10 ancestry PCs. Star = significant association of polygenic score with CP status after p-value adjustment for eight comparisons. **C)** Area under the receiver operating characteristics curve for each polygenic score, or all eight polygenic scores in the same model. Star = AUC significantly >0·5, after p-value adjustment for nine comparisons. **D)** Variance explained in CP on the liability scale attributable to each polygenic score, or to all eight polygenic scores in the same model. Population prevalence of 0·0014 was used for the Australian cohort (1 in 700 live with CP in Australia) and 0·0032 used for the MyCode cohort (1 in 313 live with CP in the U.S.). Variance on the liability scale explained significantly >0 after p-value adjustment for nine comparisons is indicated by ∗. Points = means; bars = 95% CIs for A–B (two-tailed Wald tests, α = 0·05), and 90% CIs for C–D (one-tailed Z-tests, α = 0·05). Dark blue circles = full Australian cohort (n_cases_ = 525, n_controls_ = 20,410), teal triangles = MyCode cohort with cases and controls matched on age and sex (n_cases_ = 322, n_controls_ = 1610).

In both cohorts, we evaluated the predictive performance of polygenic scores (PGS) for CP and seven related traits; age at walking onset (motor development), predisposing factors traditionally considered environmental (birth weight, gestational duration, stroke), and comorbidities (autism spectrum disorder (ASD), epilepsy, and educational attainment (EA) as a proxy for intellectual disability). Performance was assessed using: 1) logistic regression adjusted for sex and 10 ancestry principal components (Supplementary Table S2, unadjusted results which are very similar in Supplementary Table S3); 2) Area under the receiver operating characteristic curve (AUC) (Supplementary Table S4); and 3) Variance in CP explained on the liability scale (R^2^_l_) (Supplementary Table S5). In the Australian cohort, primary analyses used the full dataset (Fig. 2), with a sensitivity analysis restricted to the youngest 525 controls (1:1 with cases) (Supplementary Figure S1). In MyCode, primary analyses used age-matched controls (Fig. 2), with the unmatched cohort included as a sensitivity analysis (Supplementary Figure S1).

The multiple-PGS model including the PGS for CP and all seven related traits provided the strongest predictive value, significantly discriminating individuals with and without CP (Australia: AUC 0·60, 90% CI lower bound (LB) 0·58, padj<0·0001 [one-tailed Z-test with Benjamini-Hochberg (B-H) correction]; MyCode: AUC 0·57, 90% CI LB 0·54, padj<0·0001 [one-tailed Z-test with B–H correction]) (Fig. 2C). It also explained the largest proportion of CP liability; 1·30% in the Australian cohort (90% CI LB 0·82%, padj<0·0001 [one-tailed Z-test with B–H correction]) and 0·78% in MyCode (90% CI LB 0·35%, padj<0·0001 [one-tailed Z-test with B–H correction]) (Fig. 2D). When all eight PGS were modelled together, their individual effects remained stable, indicating largely independent contributions (Fig. 2B).

Examining individual scores, the PGS for CP and age at walking onset (a proxy for motor development) both showed minimal predictive value. Neither was significantly associated with CP nor improved discrimination beyond chance. Although each explained only a very small proportion of CP liability, it was significantly greater than zero: CP (Australia: 0·021%, MyCode: 0·050%), age at walking onset (Australia: 0·017%; MyCode: 0·045%).

Polygenic scores for known CP predisposing factors generally showed modest predictive performance, with some cohort differences. In MyCode, there was a non-significant trend of higher birthweight PGS associated with increased chance of CP (OR 1·11, 95% CI 0·99–1·24, padj = 0·58 [two-tailed Wald test with B–H correction]), and birthweight PGS discriminated CP status but did not survive multiple-testing correction (AUC 0·53, 90% CI LB 0·502, padj = 0·092 [one-tailed Z-test with B–H correction]). Birthweight PGS explained a small but significantly non-zero proportion of CP liability in MyCode (0·13%). However, in the Australian cohort, birthweight PGS was not associated with CP, did not discriminate CP status, and only explained a very small amount of CP liability (0·019%).

Gestational duration PGS showed the opposite trend; a non-significant negative association with CP in both cohorts (Australia: OR 0·93, 95% CI 0·85–1·02, padj = 0·8 [two-tailed Wald test with B–H correction], MyCode: OR 0·93, 95% CI 0·83–1·05, padj = 1 [two-tailed Wald test with B–H correction]). The PGS significantly discriminated CP status in the Australian cohort (AUC 0·53, 90% CI LB 0·51, padj = 0·03 [one-tailed Z-test with B–H correction]), but not in MyCode. Gestational duration PGS explained a small but significant portion of CP liability in both cohorts (Australia: 0·071%, MyCode: 0·078%). Overall, genetic predisposition toward higher birthweight may modestly increase the chance of CP, whereas predisposition toward longer gestation may slightly decrease this chance, although these effects remain small and uncertain, with detectability varying across cohorts.

Stroke PGS also showed a modest effect. It was positively associated with CP in the Australian cohort, though not significant after multiple testing correction (OR 1·12, 95% CI 1·03–1·23, padj = 0·06 [two-tailed Wald test with B–H correction]), with a similar non-significant trend in MyCode (OR 1·10, 95% CI 0·98–1·24, padj = 0·67 [two-tailed Wald test with B–H correction]). Discrimination was significant in the Australian cohort (AUC 0·53, 90% CI LB 0·51, padj = 0·04 [one-tailed Z-test with B–H correction]) but not in MyCode (AUC 0·53, 90% CI LB 0·51, padj = 0·079 [one-tailed Z-test with B–H correction]). Stroke PGS explained 0·14% and 0·12% of CP liability in the Australian and MyCode cohorts, respectively. Thus, individuals with higher genetic liability to stroke may have a modestly increased chance of CP.

Of the CP comorbidities, PGS for EA showed the strongest signal, though only in the Australian cohort; EA PGS was negatively associated with CP (OR 0·72, 95% CI 0·66–0·78, padj<0·0001 [two-tailed Wald test with B–H correction]), improved discrimination beyond chance (AUC 0·59, 90% CI LB 0·57, padj<0·0001 [one-tailed Z-test with B–H correction]), and explained 1·04% CP liability, the largest among all traits tested. In MyCode, however, there was no association, discrimination was no better than chance, and EA PGS only explained 0·06% of CP liability. In our sensitivity analysis of the unmatched MyCode cohort, EA PGS showed a stronger predictive performance (Supplementary Figure S1). As age-matching was not possible in the Australian cohort, we restricted controls to the youngest individuals, which yielded results consistent with the full cohort (Supplementary Figure S1). Together, these analyses suggest that the predictive performance of EA PGS for CP status may be partially or wholly explained by age-related bias.

PGS for ASD showed a modest effect in MyCode but not the Australian cohort. In MyCode, it was positively associated with CP (OR 1·14, 95% CI 1·02–1·28, padj = 0·16 [two-tailed Wald test with B–H correction]) and discriminated CP status (AUC 0·54, 90% CI LB 0·51, padj = 0·074 [one-tailed Z-test with B–H correction]), though neither remained significant after multiple testing correction. ASD PGS explained 0·019% and 0·19% of CP liability in the Australian and MyCode cohorts, respectively. In contrast, the epilepsy PGS showed no association or discrimination for CP, explaining only minimal but statistically significant amounts of CP liability (Australia: 0·031%, MyCode: 0·059%).

**Fig. 3:**
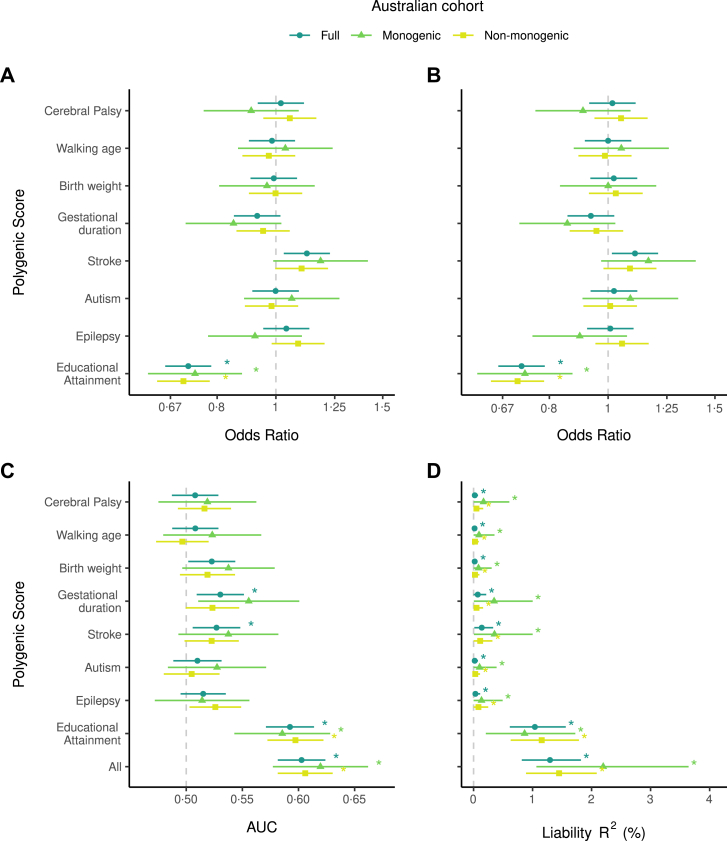
**Ability of polygenic scores for CP and seven related traits to predict cerebral palsy (CP) in the full Australian cohort and subgroups with or without a monogenic CP diagnosis. A)** Single-PGS logistic regressions, adjusted for sex and 10 ancestry PCs. Star = significant association of polygenic score with CP status after p-value adjustment for eight comparisons. **B)** Multiple-PGS logistic regression, adjusted for sex and 10 ancestry PCs (all eight polygenic scores in the same model). Star = significant association of polygenic score with CP status after p-value adjustment for eight comparisons. **C)** Area under the receiver operating characteristics curve for each polygenic score, or all eight polygenic scores in the same model. Star = AUC significantly >0·5, after p-value adjustment for nine comparisons. **D)** Variance explained in CP on the liability scale attributable to each polygenic score, or to all eight polygenic scores in the same model. Population prevalence of 0·0014 used for the Australian cohort (1 in 700 live with CP in Australia). Variance on the liability scale explained significantly >0 after p-value adjustment for nine comparisons is indicated by ∗. Points = means; bars = 95% CIs for A–B (two-tailed Wald tests, α = 0·05), and 90% CIs for C–D (one-tailed Z-tests, α = 0·05). Dark green circles = Full Australian cohort (n_cases_ = 525, n_controls_ = 20,410), lighter green triangles = individuals with a monogenic diagnosis of CP (n_cases_ = 122, n_controls_ = 20,410), lightest green squares = individuals without a monogenic diagnosis for CP (n_cases_ = 395, n_controls_ = 20,410).

Our stratified analyses demonstrated that associations, discrimination, and the amount of CP liability explained by each PGS were largely consistent when using the full Australian cohort and when stratifying the Australian cohort by presence or absence of a monogenic CP diagnosis (Fig. 3, Supplementary Tables S2–5).

## Discussion

The combination of environmental and genetic factors contributing to cerebral palsy (CP) aetiology varies between individuals and contributes to its phenotypic variability. Sequencing studies over the last decade show that rare genetic variants of large effect size (monogenic causes) can explain up to one-third of CP.^11^ Epidemiological evidence suggests that common variants also contribute, showing familial patterns of CP recurrence not explained by Mendelian inheritance.^8^, ^9^, ^10^ Most studies of common variants in CP have been small, targeted association analyses, yielding conflicting results.^14^ Here, we leveraged large, well-powered genome-wide association studies (GWAS) of CP-related traits (comorbidities and predisposing factors) to demonstrate the polygenic basis of CP. We found that combined polygenic scores (PGS) significantly discriminated CP status and we showed a quantifiable polygenic contribution to CP. Notably, associations were observed regardless of monogenic diagnosis, suggesting that common variants contribute broadly to CP susceptibility. Overall our findings support a multifactorial model of CP and highlight the long-term potential of polygenic information to complement rare variants and clinical data in guiding earlier diagnosis and intervention.

To quantify the polygenic contribution to CP, we examined variance explained by CP-specific and related-trait PGS. SNP-based heritability (h^2^_SNP_) measures the contribution of common variants to a trait, representing the upper bound of variance explained by PGS. For CP, h^2^_SNP_ has not yet been estimated. The CP-specific PGS alone explained <0·1% of CP liability, likely reflecting the limited power of the largest available CP GWAS, a FinnGen and UK Biobank meta-analysis (effective sample size N_eff_ = 3465). Combining CP and seven related-trait PGS increased variance explained to ∼1%, indicating that CP aetiology partly reflects the cumulative effect of many small-effect common alleles, consistent with a polygenic architecture. Experience from other complex traits show that GWAS sample sizes in the tens of thousands are typically required before PGS reach meaningful predictive accuracy. For example, in autism spectrum disorder (ASD), which is comorbid in 8–30% of individuals with CP, early GWAS analysing ∼1400 ASD probands in a family-based design yielded PGS explaining <1% of variance.^40^^,^^41^ The recent, largest ASD GWAS (N_eff_ = 44,367; h^2^_SNP_ = 11·8%), produced an ASD PGS explaining 2·45% of risk, increasing to 3·77% with a multi-trait PGS.^21^ Thus, larger CP GWAS of comparable scale will likely be required to substantially improve PGS accuracy and enable estimation of h^2^_SNP_, highlighting the need for international CP GWAS efforts.

All examined PGS explained some variance in CP liability, with each score contributing largely independently. For several traits this variance was very small and only a subset of PGS significantly discriminated CP status. Non-significant results could reflect limited power of smaller discovery GWAS rather than absence of effect. The predictive ability of related-trait PGS may act through two complementary mechanisms: capturing the co-occurrence of these traits with CP, and reflecting shared biology between CP susceptibility and its comorbidities or predisposing factors. Importantly, PGS for predisposing factors traditionally considered environmental (birth weight, gestational duration, stroke) showed modest predictive signal, underscoring that CP aetiology cannot be understood solely by environmental or monogenic models. Larger CP GWAS could clarify these relationships using methods such as genetic correlation, polygenic overlap, and Mendelian Randomisation, providing insight into causal pathways and mechanisms leading to CP.

Common variants associated with thrombophilia, ischaemic stroke and inflammation have been the focus of many targeted genetic association studies in CP to date.^14^ In support of previous associations, the stroke PGS was positively associated with CP and discriminated CP status. While most GWAS participants used to construct this PGS were adults with later-life stroke, several implicated genes also confer neonatal stroke risk. For example, *F5* for thrombophilia and *COL4A1*/*COL4A2* for periventricular leukomalacia and ischaemic stroke.

The opposing associations of CP with birth weight and gestational duration, and their variation across cohorts, were unexpected. In MyCode, higher birth weight PGS showed a trend toward increased CP polygenic score and modest discrimination. By contrast, gestational duration PGS significantly discriminated CP status in the Australian cohort, with trends in both cohorts suggesting that genetic predisposition for a shorter gestational duration may increase the chance of CP. This may reflect the bimodal distribution of CP incidence across gestational age, which is highest among pre-term (<37 weeks) and late-term (>42 weeks) births.^42^ Potential cohort differences may also contribute; the Australian cohort is biased toward pre-term births (35·2 ± 5·3 weeks), potentially strengthening associations with low gestational duration PGS. Whereas, if MyCode were enriched for late-term births (data unavailable), this could strengthen the association with high birth weight PGS. Additionally, the birth weight GWAS used to generate the PGS was adjusted for gestational duration in fewer than 15% of participants,^26^ which may reduce sensitivity. Properly testing CP associations may require mutual adjustment for both traits.

The PGS for educational attainment (EA) was the strongest predictor in the Australian cohort, with lower scores (i.e., a genetic profile linked to lower EA) associated with increased chance of CP. This may reflect shared genetic liability between CP and cognition, given the high prevalence of intellectual disability in CP.^3^ Alternatively, EA PGS may also capture genetic influences on neurodevelopmental or motor pathways that indirectly affect education, such as physical impairments limiting education access. The apparent strength of this association likely reflects the large sample size and power of the EA GWAS rather than uniquely strong overlap with CP. In MyCode, EA PGS showed no association in the age-matched cohort but was strongly associated in the unmatched cohort, where CP participants were younger than controls. This discordance suggests possible recruitment bias; individuals with lower EA PGS may be underrepresented in older cohorts but not younger ones. In the Australian cohort, the strong association of low EA PGS with CP persisted in both the full cohort and when restricting controls to the youngest participants, suggesting any recruitment bias may be smaller or reflect a threshold effect.

Analyses stratifying the Australian cohort by monogenic status showed similar associations, suggesting polygenic influences contribute regardless of rare variant presence. Rather than diminishing the importance of monogenic or environmental factors, these findings highlight that common variants add to the overall liability within a multifactorial landscape. This has methodological and clinical implications; future studies should model rare and common variants together rather than treating them as mutually exclusive. The demonstration here of a fundamental contribution of common variants to CP suggests future applications. An early and accurate CP diagnosis can be made from combining the Hammersmith infant neurological examination, foetal brain MRI and the absence of fidgety movements using general movements assessment however this approach does not predict comorbidities or cognitive outcomes.^18^^,^^43^ Inclusion of both monogenic and polygenic results into these models could aid in refining outcome predictions, for example in familial monogenic epilepsy a higher polygenic score is correlated with more severe seizure phenotypes.^44^ Any clinical application of this research will require adequate scoping studies, community engagement and co-designed clinical trials.^45^ More broadly, these results underscore the need to move beyond binary distinctions between ‘genetic’ and ‘environmental’ CP and toward integrated frameworks capturing the full spectrum of aetiologies.

Our study should be interpreted considering some limitations. First, a major limitation is that analyses were restricted to individuals of European genetic ancestry, which limits the generalisability of our findings. Polygenic scores are known to perform poorly outside the ancestry in which the discovery GWAS were conducted, and thus our results cannot be assumed to extend to populations of non-European ancestry, including admixed populations. Future work must prioritise the inclusion of diverse ancestries to ensure that existing health disparities are not exacerbated. This could be achieved through international collaboration and investing in underrepresented populations. Furthermore, studies should account for ancestry differences, including variations in diagnostic criteria for CP and comorbidities such as intellectual disability across countries. Second, the limited power of current CP GWAS constrains the precision of CP-specific PGS. Our findings support the need for a larger, well-powered GWAS to refine polygenic score estimates, identify biological pathways, and reveal potential therapeutic targets. Third, selection of participants from our existing biobanks and available genotyping data as convenience cohorts may have influenced the results. Our sensitivity analyses identified age differences between people with and without CP impacted the magnitude of the effect of some scores, particularly EA PGS, which may be affected by recruitment bias. Other neurodevelopmental traits and CP predisposing factors that present concurrently, or prior to diagnosis of CP were less affected by age differences. Conversely, recruitment of predominantly adults with CP in the MyCode cohort may also have influenced the results due to survivor bias excluding individuals with early mortality which is more frequent in people with severe CP compared to the general population.^46^ This is why we performed age matching with the MyCode cohorts however the effect of age matching with paediatric cohorts is unknown. Future analysis of matched paediatric case and control cohorts will be required to resolve these limitations. Finally, individuals with CP in MyCode relied on ICD-9 and 10 codes extracted from the EHR, so some individuals may have been missed if non-CP codes (e.g., spastic hemiplegia 342·1 and G81·1 in ICD9 and 10 respectively) were consistently used. We minimised this bias using a large control sample and exclusion of all patients seen by the Department of Developmental Medicine at Geisinger.

Overall, this study broadens understanding of CP aetiology and highlights the potential for integrating polygenic scores with rare variants and clinical data to enhance clinical decision-making, improving early diagnosis, intervention, and outcomes for people living with CP.

## Contributors

The initial concept for this study was conceived by MAC. JTT, ASFB, MTO, ATH, NGM, DHL, CLvE, JG, SMM, BLM, and MAC provided input to study design. Collection and processing of data was carried out by the following people for each cohort: Australian CP Biobank = JGB, AHM, CLvE, MAC, QSkin = CMO, DCW, MyCode = ASFB, MTO, DHL, SMM, RIT. Genotype data quality control and imputation for the Australian cohort was conducted by SDG. GWAS summary statistics were downloaded and formatted by JTT. The pipeline for polygenic score construction was created by SDG and BLM, and BLM constructed all polygenic weights as well as polygenic scores for the Australian cohort. JTT converted genome builds and ASFB constructed polygenic scores for MyCode. The scripts for all statistical analyses were written by JTT. Generation of final cohort data for analysis, including removal of related individuals, and all statistical analyses were carried out by JTT (Australian cohort) and ASFB (MyCode). Writing of the manuscript was led by JTT with contributions from MAC, ASFB, and BLM. Critical review of the manuscript was provided by MTO, DHL, NGM, AHM, CLvE, JG, and SMM, and all authors provided comments on the final manuscript and had final responsibility for the decision to submit for publication. JTT, SDG, NGM, and BLM had full access to the Australian cohort data, and ASFB, MTO to the MyCode data. JTT and BLM (Australian cohort), and ASFB and MTO accessed and verified the data. Supervision was provided by NGM and BLM (Australian cohort), and MTO, DHL, and SMM (MyCode), with overall coordination of the project by MAC. All authors read and approved the final version of the manuscript.

## Data sharing statement

Requests for genotyping data from the Australian CP Biobank should be made to the corresponding author and require the approval of the Women's and Children's Health Network Human Research Ethics Committee. All source data for figures provided in Supplementary Tables. Code used to conduct analyses presented in this manuscript can be found at Github (https://github.com/jodithea/Polygenic_scores_association_with_Cerebral_Palsy), which has been archived on Zenodo and assigned a https://doi.org/10.5281/zenodo.17510000 (https://doi.org/10.5281/zenodo.17510000).

## Declaration of interests

DHL serves as scientific consultant to Nest Genomics and MyOme, Inc. CMO received consulting fees from the New Zealand Public Health Agency and New Zealand Cancer Control Agency and payment for expert testimony from Maurice Blackburn Lawyers for matters pertaining to skin cancer in all cases. DCW received consulting fees from the Melanoma Network New Zealand (MelNet) and Cancer Institute NSW; Payments for presentations from the New Zealand Cancer Control Agency and Melanoma Institute Australia; Payment for travel support from the European Dermato-oncology Association; participated in an unpaid capacity on the data safety monitoring board of the Princess Alexandra Hospital and board of the Sax Institute. All other authors declare no competing interests.
