# Supplementary material for: Testing the performance of polygenic scores for multiple traits to explain cerebral palsy in two independent cohorts
Source: eBioMedicine. 2026 Mar 14;126:106208. doi: 10.1016/j.ebiom.2026.106208 (PMC13000708; doi:10.1016/j.ebiom.2026.106208)
Supplement: Supplementary Information [file mmc1.pdf]

Supplementary Material

Testing the performance of polygenic scores for multiple traits to explain cerebral palsy in two independent cohorts

Jodi T Thomas PhD[1,2], Alexander SF Berry PhD[3], Matthew T Oetjens PhD[3], Jesia G Berry PhD[4], Prof Alastair H MacLennan MD[4], Scott D Gordon PhD[1], Andrew T Hale MD PhD[5, 6], Catherine M Olsen PhD[7], Prof David C Whiteman MBBS PhD[7], Rebecca I Torene PhD[3], Prof David H Ledbetter PhD[8], Prof Nicholas G Martin PhD[1], Clare L van Eyk PhD[4], Prof Jozef Gecz PhD[4,9], Prof Scott M Myers MD[3], Brittany L Mitchell PhD[1,2,10], Mark A Corbett PhD[4]

[1]Brain and Mental Health Program, QIMR Berghofer, Brisbane, QLD, Australia
[2]School of Biomedical Sciences, The University of Queensland, Brisbane, QLD, Australia
[3]Department of Developmental Medicine, Geisinger College of Health Sciences, Lewisburg, PA, USA
[4]School of Medicine and Robinson Research Institute, College of Health, Adelaide University, Adelaide, SA, Australia
[5]Department of Neurosurgery, The University of Alabama at Birmingham, Birmingham, AL, USA.
[6]Neuroscience Institute, University of Cape Town, Cape Town, South Africa
[7]Population Health Program, QIMR Berghofer, Brisbane, QLD, Australia
[8]Institute for Pediatric Rare Diseases, College of Medicine, Florida State University, Tallahassee, FL, USA
[9]South Australian Health and Medical Research Institute, Adelaide, SA, Australia
[10]School of Biomedical Sciences, Faculty of Health, Queensland University of Technology, Brisbane, QLD, Australia

Contents

**Supplementary Text A.** Procedures: Genotyping and Quality Control, Polygenic Scores.	Page 2
**Supplementary Text B.** Sensitivity Analyses.	Page 3
**Supplementary Table 1.** Sample characteristics for both cohorts included in the sensitivity analyses to assess potential age biases.	Page 4
**Supplementary Table 2.** Results from single-PGS and multiple-PGS logistic regression models adjusted for sex and 10 ancestry principal components (i.e. one polygenic score as the predictor or all eight polygenic scores simultaneously in the one model).	Page 5
**Supplementary Table 3.** Results from unadjusted single-PGS and multiple-PGS logistic regression models (i.e. one polygenic score as the predictor or all eight polygenic scores simultaneously in the one model).	Page 5
**Supplementary Table 4.** Results from receiver operating characteristic analysis conducted for each polygenic score individually and for all polygenic scores combined.	Page 5
**Supplementary Table 5.** The variance in cerebral palsy (CP) on the liability scale that is explained by each polygenic score individually, or all polygenic scores combined.	Page 5
**Supplementary Figure 1.** Ability of polygenic scores for cerebral palsy (CP) and seven related traits to distinguish between individuals with and without CP in two independent cohorts, with sensitivity analyses to determine the effect of age-related biases.	Page 6

Supplementary Text A

Procedures

Genotyping and Quality Control

Australian cohort. Participants were genotyped using Illumina GSA v1 and v3. Sex was inferred from genotype data, with XX individuals as female and XY as male. Specifically, individuals were classified as female if X-chromosome heterozygosity ≥0.25 and Y-chromosome genotype calls ≤105, and as male if X-chromosome heterozygosity ≤0.085 and Y-chromosome genotype calls ≥175. Self-reported sex (male/female) was collected in the Australian CP Biobank, and for QSkin was obtained from the Australian Electoral Roll. Quality control procedures removed individuals (after investigation) with call rate <98%, discordance between genetically inferred and reported sex, or evidence of spurious relatedness to multiple samples. Samples were merged with the 1000 Genomes Phase 3 project samples[1] and genetic principal components (PCs) were calculated in 'smartpca' (build 13050)[2] using a thinned set of single nucleotide polymorphisms (SNPs) shared with the reference genotypes (262,506 SNPs). The 1000 Genomes individuals were used to define the PC axes. Participants without genetic similarity to European reference groups (>6 standard deviations (SD) from Ancestry PCs PC1/PC2 centroid for those populations) were excluded. Pre-imputation marker quality control was done using GenomeStudio and PLINK v1.9[3,4] and custom scripts, removing SNPs with a minor allele frequency < 0·01, SNP call rate < 95%, GenTrain < 0·6, or deviating from Hardy-Weinberg equilibrium ($p < 1 \times 10^{-6}$). Map and strand alignment was based on BLAST searches of the marker primer sequences relative to hg19, as carried out by Will Rayner (https://www.strand.org.uk/). Imputation was then done using the Haplotype Reference Consortium 1.1 reference panel[5] on the Michigan Imputation Server[6], restricted to markers passing quality control in all batches (N = 441,530). After quality control, there was no missing data; CP status was available for all participants, and all other variables were derived from the genotype data (polygenic score, sex, and ancestry principal components).

MyCode. Participants were genotyped using the Illumina GSA-24v1, GSA-24v2, and OmniExpressExome-8 BeadChip. Array data were filtered to remove SNPs with minor allele frequency < 0·01, Hardy-Weinberg equilibrium $p < 1 \times 10^{-15}$, missingness > 1%. The dataset was phased using eagle v2.4[7] and imputed using MINIMAC4[8] against 97,256 deeply sequenced genomes in the TOPMed reference panel using the TOPMed Imputation Server.[9,10] The first 20 principal components (PCs) were calculated using PLINK v1.9 by projecting each participant onto PCs derived from individuals in the 1000 Genomes Project Phase 3 representing European, African, and East Asian continental ancestry populations. Individuals from the "Americans of African Ancestry in SW USA" and "African Caribbean in Barbados" populations were excluded from the reference panel. PC projection was performed using a set of 25,189 autosomal variants that met the following criteria: present in both the 1000 Genomes Project and MyCode datasets, minor allele frequency (MAF) > 0·01, not located in regions of long-range linkage disequilibrium (LD) or in genomic regions under recent selection[11,12], and not ambiguous (e.g., A/T or C/G SNPs). These PCs were subsequently used for ancestry estimation using Rye.[13] Individuals with >50% estimated ancestry from one of the three continental populations were assigned to that ancestry group. Participants who did not meet this threshold for any single group were classified as Admixed/Other. Only individuals of European ancestry were included in our analyses here. Sex was determined based on EHR-documented information. Individuals were excluded from analyses if they lacked sex data or had no recorded EHR encounters.

Polygenic Scores

Genome-wide association study (GWAS) summary statistics for CP and seven related traits were first quality-controlled to remove variants with minor allele frequency (MAF) < 0·01 and imputation INFO score < 0·6 (if available). Genome build conversion was performed as required, prioritising rsID matching and otherwise using CrossMap (v0.6.1)[14] with NCBI dbSNP 155 patch 13. These summary statistics were then used to create polygenic weights using SBayesRC in GCTB (v2.5.2)[15], a Bayesian regression framework that models linkage disequilibrium (LD) between SNPs while incorporating functional annotation information to improve effect size estimation. Analyses used the provided LD eigen-decomposition data from UK Biobank participants of European ancestry (7 million imputed SNPs), the provided per-SNP functional annotations (7 million SNPs), a chain length of 21,000 with a burn-in of 1,000, and the remaining default settings. The posterior SNP effect sizes estimated by SBayesRC were then used to calculate polygenic scores (PGS) using individual genotype data from each target cohort. PLINK (v1.9)[4] was used to construct PGS, summing the number of effect alleles weighted by their posterior mean effect estimates (using the flag --score).

Supplementary Text B

Sensitivity Analyses

As a sensitivity analysis to assess potential age bias, as age-matching was not possible in the Australian cohort we repeated analyses using only the youngest 525 controls (1:1 ratio with cases). The age difference between participants with CP and controls was still significant, although smaller (mean difference = 26·42 years, 95% CI 25·72–27·14, t = 72·75, df = 976·22, p<0·0001 [two-tailed T-test]). The proportion of females was also still lower among individuals with CP (40·0%) compared with controls (80·2%) (χ^2 = 175·140, df = 1, p<0·0001 [Chi-squared test]) (Supplementary Table 1).

For the sensitivity analysis in MyCode, we examined the full, unmatched cohort (322 individuals with CP and 129,628 controls), in which participants with CP were significantly younger than controls (mean difference = 7·73 years, 95% CI 15·45–20·01, t = 15·31, df = 322·15, p<0·0001 [two-tailed T-test]). The proportion of females was lower among individuals with CP (51·86%) than controls (60·07%) (χ^2 = 8·67, df = 1, p=3·23 × 10^{-3} [Chi-squared test]) (Supplementary Table 1).

Supplementary Table 1. Sample characteristics for both cohorts included in the sensitivity analyses to assess potential age biases. In the Australian cohort, age-matching was not possible, so we restricted controls to the youngest 525 (1:1 with cases). As results were similar, the full cohort is reported as primary and the restricted set as a sensitivity analysis presented here. In MyCode, age- and sex-matched analyses are reported as primary, with the unmatched cohort as a sensitivity analysis presented here. Age = age at time of survey (Australia) or age at last electronic health record retrieval (MyCode), CP = cerebral palsy, SD = standard deviation. Note that only the characteristics of controls has changed, as the same participants with CP are used in the primary and sensitivity analyses. Note that sex is the only variable from this table included in the analyses, all other variables are provided for descriptive purposes.

Characteristic	Australian Cohort		MyCode	
	**Control**	**CP**	**Control**	**CP**
**Sex**				
Female (N (%))	421 (80·2%)	210 (40·0%)	77,865 (60·1%)	167 (51·9%)
Male (N (%))	104 (19·8%)	315 (60·0%)	51,763 (39·9%)	155 (48·1%)
Unknown (N (%))	0 (0%)	0 (0%)	0 (0%)	0 (0%)
**Age (years)**				
Mean ± SD (range)	35·3 ± 6·3 (18-44)	8·9 ± 5·1 (1-43)	58·3 ± 17·7 (0·3-90·1)	40·6 ± 20·8 (1·7-89·1)
Unknown (N (%))	0 (0%)	64 (12·2%)	0 (0%)	0 (0%)

Supplementary Table 2. Results from single-PGS and multiple-PGS logistic regression models adjusted for sex and 10 ancestry principal components (i.e. one polygenic score as the predictor or all eight polygenic scores simultaneously in the one model). Results are shown for both the Australian and MyCode cohorts, and for the analysis in which the Australian cohort was stratified based on the presence or absence of a monogenic diagnosis for cerebral palsy (CP). PGS = polygenic score, OR = odds ratio, SE = standard error, Z = two-sided Z statistic, P = p-value, Padj = p-value adjusted for eight comparisons using the Benjamini-Hochberg method, CI = confidence interval.

This table is too large to fit here and is thus in the file 'Supplementary_Tables.xlsx.'

Supplementary Table 3. Results from unadjusted single-PGS and multiple-PGS logistic regression models (i.e. one polygenic score as the predictor or all eight polygenic scores simultaneously in the one model). Results are shown for both the Australian and MyCode cohorts, and for the analysis in which the Australian cohort was stratified based on the presence or absence of a monogenic diagnosis for cerebral palsy (CP). PGS = polygenic score, OR = odds ratio, SE = standard error, Z = two-sided Z statistic, P = p-value, Padj = p-value adjusted for eight comparisons using the Benjamini-Hochberg method, CI = confidence interval.

This table is too large to fit here and is thus in the file 'Supplementary_Tables.xlsx.'

Supplementary Table 4. Results from receiver operating characteristic analysis conducted for each polygenic score individually and for all polygenic scores combined. Results are shown for both the Australian and MyCode cohorts, and for the analysis in which the Australian cohort was stratified based on the presence or absence of a monogenic diagnosis for cerebral palsy (CP). AUC = area under the receiver operating characteristic curve, SE = standard error, Z = one-sided Z statistic for AUC > 0.5, P = p-value, Padj = p-value adjusted for nine comparisons using the Benjamini-Hochberg method, CI = confidence interval. 90% CI is displayed because a one-tailed Z-test was used.

This table is too large to fit here and is thus in the file 'Supplementary_Tables.xlsx.'

Supplementary Table 5. The variance in cerebral palsy (CP) on the liability scale that is explained by each polygenic score individually, or all polygenic scores combined. Results are shown for both the Australian and MyCode cohorts, and for the analysis in which the Australian cohort was stratified based on the presence or absence of a monogenic diagnosis for CP. R2l = variance in CP on the liability scale (as a proportion, multiply by 100 for a percentage), SE = standard error, Z = one-sided Z statistic for $R^2l > 0$, P = p-value, Padj = p-value adjusted for nine comparisons using the Benjamini-Hochberg method, CI = confidence interval. 90% CI is displayed because a one-tailed Z-test was used.

This table is too large to fit here and is thus in the file 'Supplementary_Tables.xlsx.'

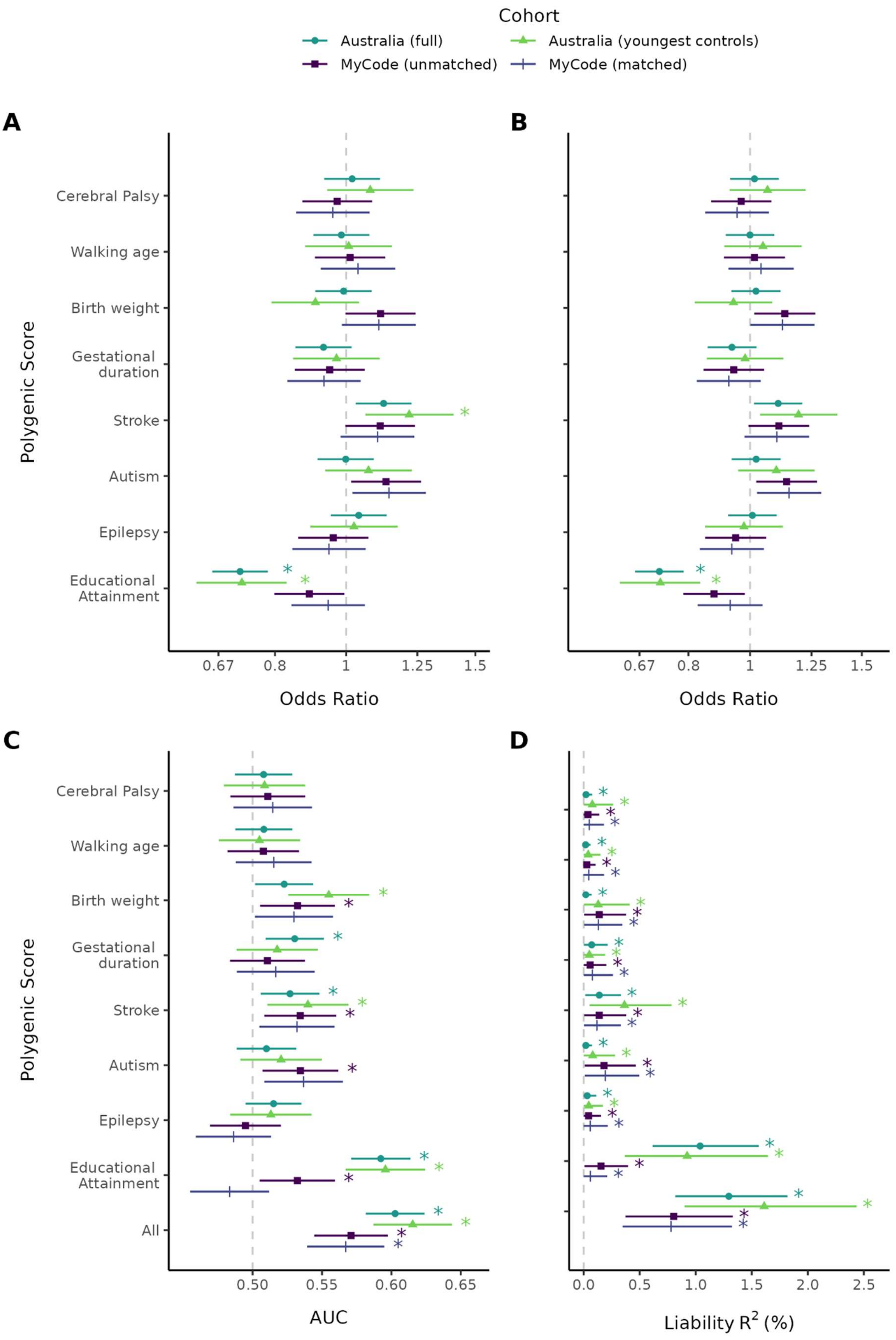

Cohort
Australia (full)
Australia (youngest controls)
MyCode (unmatched)
MyCode (matched)
A
B
C
D
Cerebral Palsy
Walking age
Birth weight
Gestational duration
Stroke
Autism
Epilepsy
Educational Attainment
All
Polygenic Score
Odds Ratio
0.67
0.8
1
1.25
1.5
AUC
0.50
0.55
0.60
0.65
Liability R^2 (%)
0.0
0.5
1.0
1.5
2.0
2.5

Supplementary Figure 1. Ability of polygenic scores for cerebral palsy (CP) and seven related traits to distinguish between individuals with and without CP in two independent cohorts, with sensitivity analyses to determine the effect of age-related biases. A) Single-PGS logistic regressions, adjusted for sex and 10 ancestry PCs (one polygenic score in each model). Star = significant association of polygenic score with CP status after p-value adjustment for eight comparisons. **B)** Multiple-PGS logistic regression, adjusted for sex and 10 ancestry PCs (all eight polygenic scores in the same model). Star = significant association of polygenic score with CP status after p-value adjustment for eight comparisons. **C)** Area under the receiver operating characteristics curve for each polygenic score, or all eight polygenic scores in the same model. Star = AUC significantly > 0·5, after p-value adjustment for nine comparisons. **D)** Variance explained in CP on the liability scale attributable to each polygenic score, or to all eight polygenic scores in the same model. Population prevalence of 0·0014 was used for the Australian cohort (1 in 700 have CP in Australia) and 0·0032 used for the MyCode cohort (1 in 313 have CP in the U.S.). Star = variance on the liability scale explained significantly > 0 after p-value adjustment with the Benjamini-Hochberg procedure for nine comparisons. Points = means; bars = 95% CIs for A–B (two-tailed Wald tests, $\alpha = 0 \cdot 05$), and 90% CIs for C–D (one-tailed Z-tests, $\alpha = 0 \cdot 05$). Dark blue circles = full Australian cohort (n_{cases} = 525, $n_{controls}$ = 21,969), lighter green triangles = Australian cohort with controls restricted to the youngest 525 individuals (1:1 case:control ratio) (n_{cases} = 525, $n_{controls}$ = 525), dark purple squares = full unmatched MyCode cohort (n_{cases} = 322, $n_{controls}$ = 129,628), blue lines = MyCode cohort with cases and controls matched on age (n_{cases} = 322, $n_{controls}$ = 1,610). (previous page).

References

1. Auton, A. *et al.* A global reference for human genetic variation. *Nature* **526**, 68–74 (2015).

2. Patterson, N., Price, A. L. & Reich, D. Population Structure and Eigenanalysis. *PLOS Genet.* **2**, e190 (2006).

3. Chang, C. C. *et al.* Second-generation PLINK: rising to the challenge of larger and richer datasets. *GigaScience* **4**, (2015).

4. Purcell, S. *et al.* PLINK: A Tool Set for Whole-Genome Association and Population-Based Linkage Analyses. *Am. J. Hum. Genet.* **81**, 559–575 (2007).

5. McCarthy, S. *et al.* A reference panel of 64,976 haplotypes for genotype imputation. *Nat. Genet.* **48**, 1279–1283 (2016).

6. Das, S. *et al.* Next-generation genotype imputation service and methods. *Nat. Genet.* **48**, 1284–1287 (2016).

7. Loh, P.-R. *et al.* Reference-based phasing using the Haplotype Reference Consortium panel. *Nat. Genet.* **48**, 1443–1448 (2016).

8. Fuchsberger, C., Abecasis, G. R. & Hinds, D. A. minimac2: faster genotype imputation. *Bioinformatics* **31**, 782–784 (2015).

9. Das, S. *et al.* Next-generation genotype imputation service and methods. *Nat. Genet.* **48**, 1284–1287 (2016).

10. Taliun, D. *et al.* Sequencing of 53,831 diverse genomes from the NHLBI TOPMed Program. *Nature* **590**, 290–299 (2021).

11. Belbin, G. M. *et al.* Toward a fine-scale population health monitoring system. *Cell* **184**, 2068-2083.e11 (2021).

12. Bycroft, C. *et al.* The UK Biobank resource with deep phenotyping and genomic data. *Nature* **562**, 203–209 (2018).

13. Conley, A. B. *et al.* Rye: genetic ancestry inference at biobank scale. *Nucleic Acids Res.* **51**, e44 (2023).

14. Zhao, H. *et al.* CrossMap: a versatile tool for coordinate conversion between genome assemblies. *Bioinforma. Oxf. Engl.* **30**, 1006–1007 (2014).

15. Zheng, Z. *et al.* Leveraging functional genomic annotations and genome coverage to improve polygenic prediction of complex traits within and between ancestries. *Nat. Genet.* **56**, 767–777 (2024).
